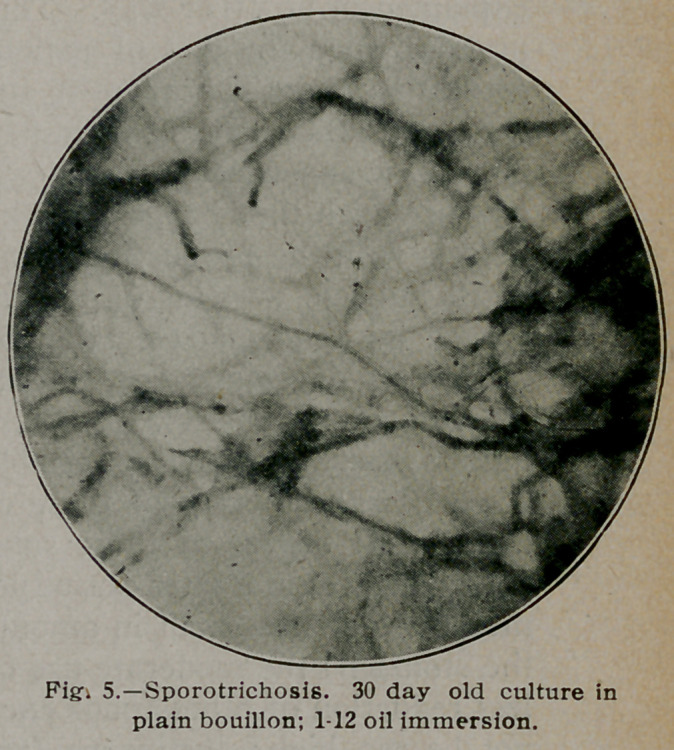# Sporotrichosis

**Published:** 1913-11

**Authors:** 


					﻿Sporotrichosis, B. W. Rhamy and W. W. Carey, Fort Wayne.
Jour, of Ind. State Med. Assn., June, 1913. Blastomycosis or
oidiomycosis was first described as a systemic infection by Gil-
christ in 1894. About 200 cases have been reported in the world’s
literature. The first author has seen two previous cases, diag-
nosed clinically. The present patient was a male, aged 27, who
gave a varied but not very significant history. His first boil
or abscess occurred in 1904 and similar lesions had occurred
intermittently ever since. Cultures were made by Henry Newton
Cole of Cleveland, who had seen about a dozen cases in Paris
and Berne. Autogenic vaccines have been used on the case
since September, 1912, apparently favoring healing but not pre-
venting the development of subsequent abscesses, the patient not
yet being well. Cuts supplied by courtesy of authors and editor.
				

## Figures and Tables

**Fig. 1. f1:**
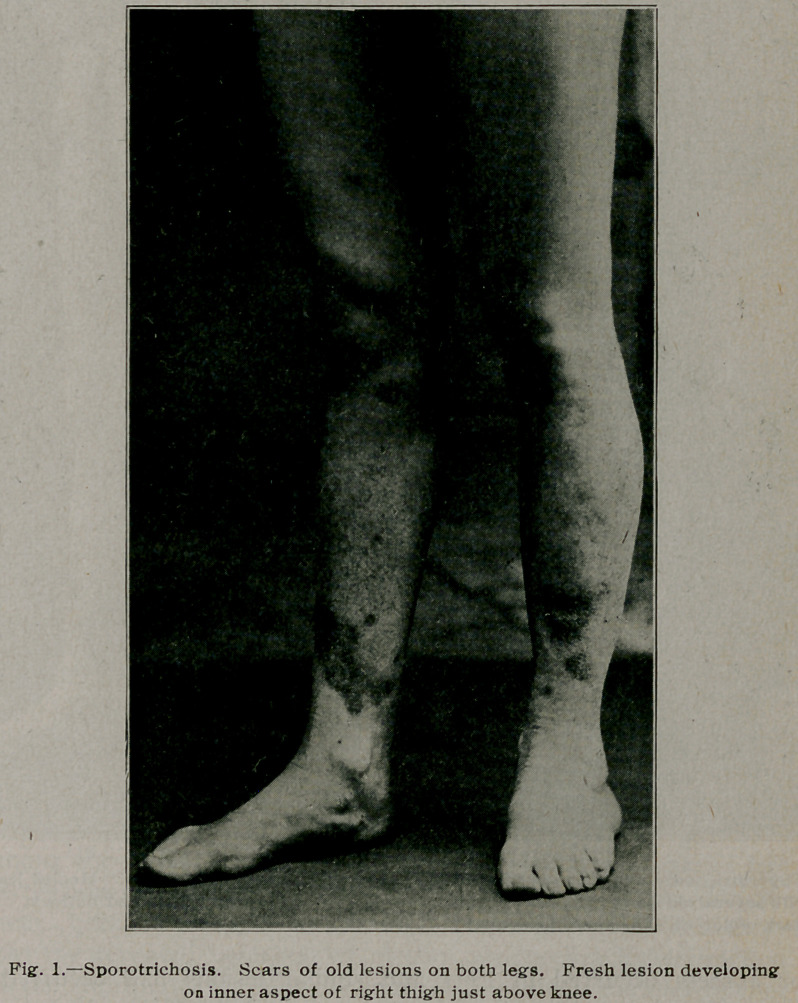


**Fig. 2. f2:**
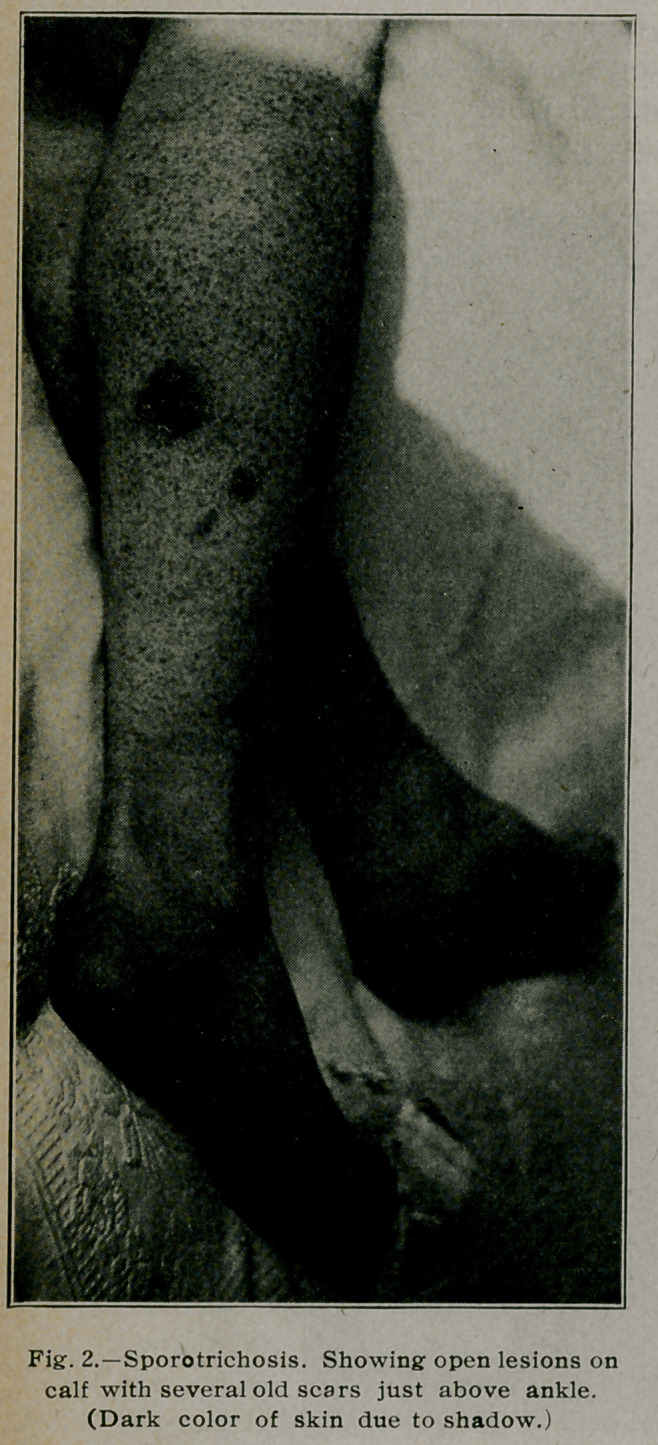


**Fig. 3. f3:**
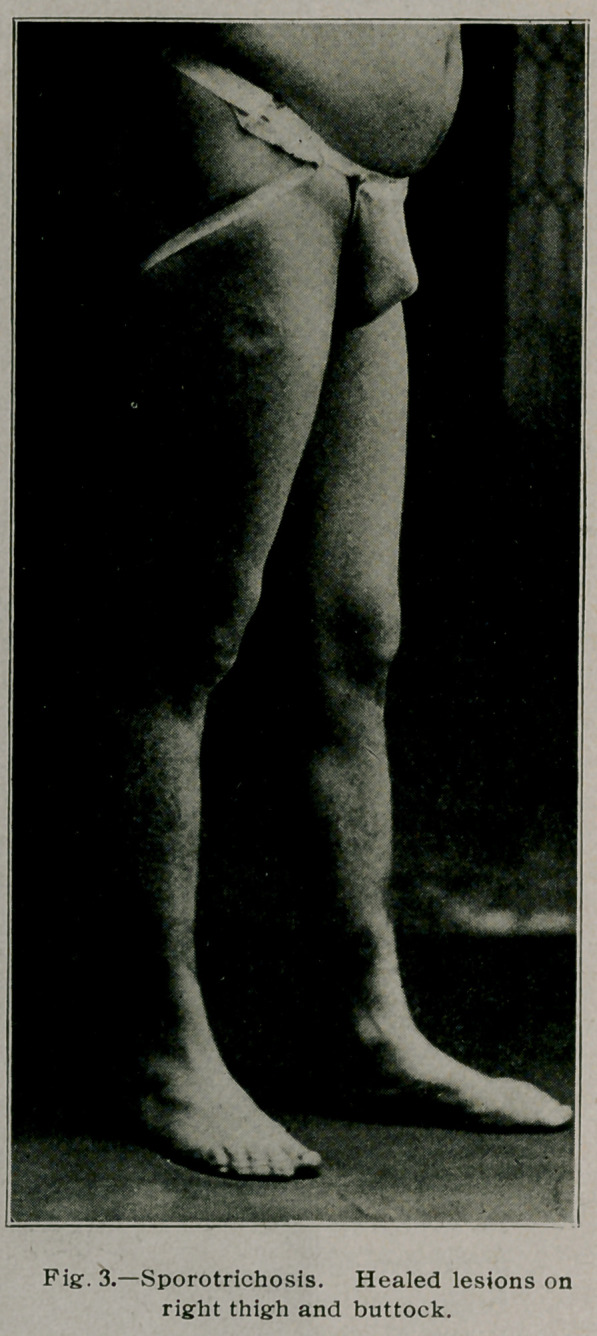


**Fig. 4. f4:**
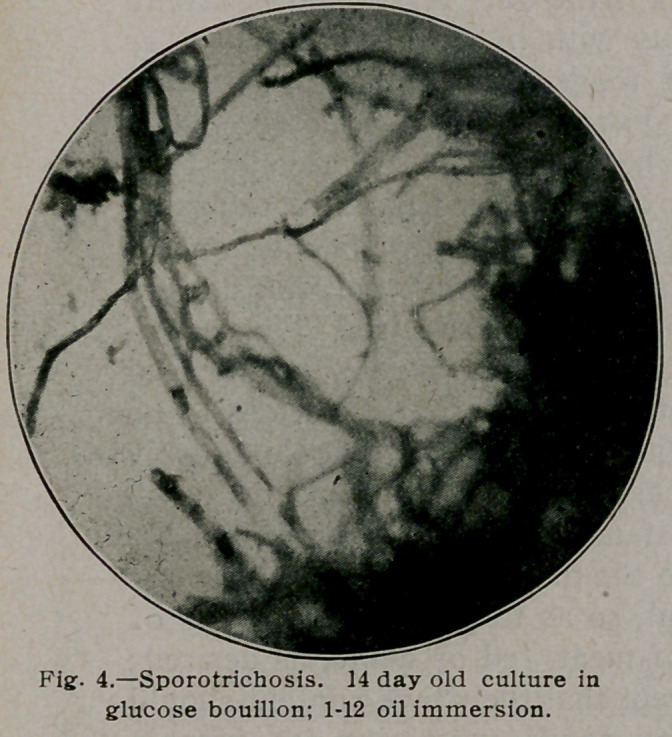


**Fig. 5. f5:**